# Exploiting of Green Synthesized Metal Oxide Nanoparticles in the Potentiometric Determination of Metformin Hydrochloride in Pharmaceutical Products

**DOI:** 10.1155/2024/8354311

**Published:** 2024-04-05

**Authors:** Shikhah Almutairi, Nawal A. Alarfaj, Adibah M. Almutairi, Maha F. El-Tohamy

**Affiliations:** Department of Chemistry, College of Science, King Saud University, P.O. Box 22452, Riyadh 11495, Saudi Arabia

## Abstract

The advanced and highly functional properties of Al_2_O_3_ and NiO nanoparticles promote the widespread use of metal oxides as remarkable electroactive materials for sensing and electrochemical applications. The proposed study describes a comparison of the sensitivity and selectivity of two modified wire membrane sensors enriched with Al_2_O_3_ and NiO nanoparticles with conventional wire membranes for the quantification of the antidiabetic drug metformin hydrochloride (MTF). The results show linear relationships of the enriched Al_2_O_3_ and NiO nanosensors over the concentration ranges 1.0 × 10^−10^–1.0 × 10^−2^ mol L^−1^ and 1.0 × 10^−6^–1.0 × 10^−2^ M for both the modified sensors and the conventional coated wire membrane sensors. The regression equations were *E*_mV_ = (52.1 ± 0.5) log (MTF) + 729 for enriched nanometallic oxides, *E*_mV_ = (57.04 ± 0.4) log (MTF) + 890.66, and *E*_mV_ = (58.27 ± 0.7) log (MTF) + 843.27 with correlation coefficients of 0.9991, 0.9997, and 0.9998 for the aforementioned sensors, respectively. The proposed method was fully validated with respect to the recommendations of the International Union of Pure and Applied Chemistry (IUPAC). The newly functionalized sensors have been successfully used for the determination of MTF in its commercial products.

## 1. Introduction

Various nanostructured metal oxides have already found wide applications. They are being researched for their promising applications in almost all scientific fields, including optics, catalysis, energy, electronics, sensors, environment, information technology, medicine, materials chemistry, biomedicine, and agriculture. These diverse applications have led scientists to develop different variants for the production of metal oxide nanoparticles with desired properties [1].

Nanomaterials play an important role in the development of chemosensors and biosensors, especially due to their distinct physical and chemical properties, such as good conductivity, surface-to-volume ratio, high mechanical strength, and excellent electrocatalytic activity [2]. Green nanotechnology is a topic of great interest in research studies around the world as it is the best way to reduce the negative impacts of nanomaterial production and use while reducing the risks of nanotechnology [3]. The principles of green synthesis can therefore be explained by a number of factors, including minimizing waste, reducing pollution, and using safer solvents. The use of plant extracts is one of the currently available green synthesis techniques for metal oxide nanoparticles (MONPs) [4].

Nowadays, the main focus is on the use of metal oxide-modified sensors for the detection and quantification of pharmaceutical compounds. The chemical properties and advanced physical characteristics of nickel oxide nanoparticles (NiONPs) and aluminum oxide nanoparticles (Al_2_O_3_NPs) promote their use in various applications [5]. The selectivity and sensitivity of ion-selective sensors are known to depend on the type of ionophore and the properties of the plasticizers and additives used. They also depend on the composition of the membrane during the manufacturing process. Nanoparticles are an excellent addition for enhancing electrode performance and reducing electrical resistance [6].

*Pennisetum glaucum* (millet) is considered to be one of the most nutritious cereals of all. It is unique among cereals in having a higher mineral content and micronutrient density than rice, wheat, barley, etc., and has outstanding nutritional properties. The richness in phytochemicals (polyphenols and fiber) increases the nutraceutical potential of millet and makes it a powerhouse of health-promoting nutrients [7]. The bioactive compounds present in millet, such as flavonoids, alkaloids, saponins, tannins, phenols, terpenoids, proteins, carbohydrates, and amino acids, have a large number of functional groups (O-H, N-H, S-H, -COOH, C=O, and C-halide) [8]. These functional groups can serve as reducing, capping, and stabilizing agents in the production of nanomaterials. The literature search revealed various reports on the use of millet extract in the synthesis of nanoparticles [9–11].

**Figure 1:**
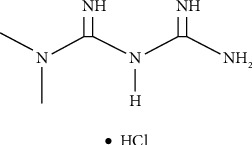
Structural formula of metformin hydrochloride.

Metformin, 1,1-dimethylbiguanide hydrochloride (Figure 1) is an oral hypoglycemic agent used in medicine for the treatment of diabetic patients [12–15]. Metformin is the most commonly prescribed drug for the treatment of type 2 diabetes (T2D) [16]. In contrast to other diabetes medications, metformin has a positive effect on body weight and has no hypoglycemic side effects. The main target of metformin is presumably the liver. The drug reaches the liver cells and suppresses the production of glucose in the liver, which leads to a reduction in blood glucose levels [17].

The literature search revealed several analytical methods for the detection of MTF. These methods are spectrophotometry [18, 19] and separation chromatography [20, 21]. A number of sensors have been developed for the determination of MTF [22], but they still have some limitations.

Potentiometry is one of the most important electrochemical techniques, and researchers have long been interested in the applications of potentiometric-based sensors [23]. Hundreds of different sensors have been developed in this field and published in the literature to date [24]. Today, potentiometric sensors are the focus of interest due to the successful development of sensors in many applications [25–27]. Due to the high demand and constant technological progress, the world of sensors is diverse and rapidly evolving. Electrochemical sensors are widely used in the food, oil, and agricultural industries as well as in environmental and biomedical applications. They offer a convenient and affordable solution for the detection of variable analytes [28–32].

The use of metal oxides as electroamplifiers in electrochemical sensors has been reported, e.g., the use of aluminum oxide and nickel oxide nanoparticles in various sensor systems. Due to their strong electrocatalytic activity, low cost, high ability to bind organics, small size, high degree of crystallinity, and large surface-to-volume ratio, metal oxide nanoparticles are widely used and have been established as an active electrocatalyst for the detection of a variety of compounds [33]. The active sites, electrochemically active surface area, surface energy, and other factors are generally closely related to the electrocatalytic properties of metal oxide nanoparticles [34]. The catalytic materials were fabricated as small as possible to increase the number of available active sites and the available surface area so that the metal oxide-based nanoparticles exhibit high sensing performance [35–38].

Undoubtedly, a formula for the development and improvement of ion-selective sensors with low detection limits, repeatability, and good chemical stability is needed. Currently, there are no known modified potentiometric sensors for the detection of metformin hydrochloride based on fabricated metal oxide nanoparticles.

The aim of the present study is to use an aqueous millet extract and a natural reduction source to synthesize Al_2_O_3_NPs and NiONPs from their precursors (aluminum nitrate and nickel sulfate) under certain optimized conditions. The synthesized metal oxide nanostructures were subsequently confirmed by various spectroscopic and microscopic investigations. Two new and sensitive potentiometric sensors modified with pre-synthesized nanoparticles were fabricated, and their efficiency in the determination of metformin hydrochloride was investigated. In addition, a comparative study between the conventional sensor and the metal oxide nanoparticle-enriched sensors was performed.

## 2. Experimental

### 2.1. Chemicals and Reagents

Pure MTF and its pharmaceutical preparation (Metfor® 500 mg/tablet) were provided by Tabuk Pharmaceutical MFG.CO. (Saudi Arabia). Tetrahydrofuran (THF) 97%, methanol 99.9%, acetone 99.9%, phosphotungstic acid (PTA), sodium hydroxide (NaOH), hydrochloric acid (HCl), polyvinyl chloride (PVC) of high molecular weight, ortho-nitrophenyl octyl ether (*o*-NPOE), aluminum nitrate (Al(NO_3_)_3_), and nickel sulfate (NiSO_4_) were acquired from Sigma-Aldrich, Hamburg, Germany.

### 2.2. Instrumentation

Potentiometric measurements were performed with a digital pH-mV (HANNA, model-211) with an Ag/AgCl reference electrode in conjunction with an indicator electrode. A pH meter (Metrohm model 744) was also used for pH measurements. UV-vis spectral analysis was performed using a UV 2450 spectrophotometer (Shimadzu Corporation, Kyoto, Japan). A scanning electron microscope (SEM) and a transmission electron microscope (TEM) (JEM-2100F, JEOL Ltd, USA) were used to study the particle size and surface morphology of Al_2_O_3_NPs and NiONPs. To determine the functional groups that might be present in the Al_2_O_3_NPs and NiONPs after fabrication, Fourier transform infrared spectroscopy (FT-IR) was performed using a Spectrum spectrometer BX (PerkinElmer, Waltham, USA).

### 2.3. Preparation of Extract *Pennisetum glaucum* (Millet)

**Scheme 1:**
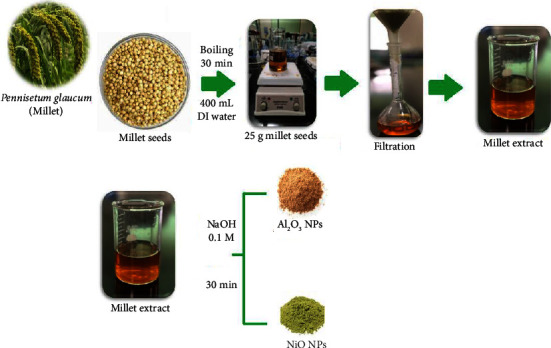
Extraction of *Pennisetum glaucum* (millet) and synthesis of Al_2_O_3_ and NiO nanoparticles.

The *Pennisetum glaucum* (millet) seeds were obtained from a local source (Riyadh, Saudi Arabia). 25 g of cleaned seeds were boiled with 400 mL of deionized water for 30 min. The content was cooled and filtered using Whatman filter paper, No. 4. The resulting extract was kept in a refrigerator for storage at 4°C (Scheme 1).

### 2.4. Optimization Conditions of Green Synthesis of Nanoparticles

The synthesis process of Al_2_O_3_ and NiO nanoparticles was performed under certain optimized conditions including the use of different volumes of the plant extract (5–30 mL), the effect of reaction time intervals (10–60 min), the suitable pH of the solution (pH = 2–12), and the effect of temperature using three different degrees (25, 35, and 55°C). The synthesis process was performed under constant stirring at 3000 rpm for 30 min. The selected conditions were 20 mL of extract and 30 min reaction time. pH was adjusted using sodium hydroxide to 11.

### 2.5. Green Synthesis of Al_2_O_3_ and NiO Nanoparticles

The synthesis of Al_2_O_3_NPs was carried out under constant stirring by 100 mL of aluminum nitrate (0.2 M) with 20 mL of millet extract subsequently. A dropwise addition of 5 mL of sodium hydroxide solution 0.1 M was made to adjust the pH to 11. Al_2_O_3_NPs started to form after the mixture had been held under magnetic stirring for 30 minutes at room temperature. To eliminate any excess sodium hydroxide, the produced nanoparticles were filtered through filter paper and then washed three times with distilled water and one time with methanol. The produced Al_2_O_3_NPs were dried for 24 hours at room temperature [39].

The synthesis of NiONPs was carried out under constant stirring by 100 mL of nickel sulfate (0.2 M) with 20 mL of millet extract subsequently. A dropwise addition of 5 ml of sodium hydroxide solution 0.1 M was made to adjust the pH to 11. NiONPs started to form after the mixture had been held under magnetic stirring for 30 minutes at room temperature. To eliminate any excess sodium hydroxide, the produced nanoparticles were filtered through filter paper and then washed three times with distilled water and one time with methanol. The produced NiONPs were dried for 24 hours at room temperature [40]. The synthesis process was previously illustrated in Scheme 1.

### 2.6. Preparation of Stock Drug Solution

A stock solution of MTF (1.0 × 10^−2^ M) was prepared by dissolving 0.13 g in 100 mL of distilled water. Serial dilutions were prepared with distilled water.

### 2.7. Preparation of Ion Pair

The ion pair (metformin-phosphotungstate) MTF-PT was prepared by mixing 50 mL of 1.0 × 10^−2^ M of MTF solution and 50 mL of 1.0 × 10^−2^ M precipitating agent (phosphotungstic acid) PTA. The obtained precipitate was filtered, properly washed with distilled water, and then fully dried overnight at room temperature [41].

### 2.8. Membrane Composition

Three different coated membranes were prepared using electroactive materials MTF-PT, MTF-PT-Al_2_O_3_, and MTF-PT-NiO nanoparticles. The conventional coated wire membrane was prepared by mixing 190 mg of PVC and 0.35 mL plasticizer *o*-NPOE and 10 mg of ion pair (MTF-PT) in 5 mL of THF. The prepared mixture was placed in a petri dish with a diameter of 3 cm and allowed to gradually evaporate there at room temperature. 5 mg of the previously synthesized nanoparticles Al_2_O_3_ and NiO was added separately to the aforementioned composition of the membrane to create the modified membranes [42].

### 2.9. Coated Wire Membrane Composition

The Al wire's tip was cleaned with distilled water and acetone and then dried. The wire was coated by swiftly dipping the wire into the coating solution multiple times and letting it dry at room temperature. The manufactured sensor was preconditioned by soaking for 24 h in a 1.0 × 10^−3^ M of MTF solution.

### 2.10. Electrode Calibration Graph

About 10 mL aliquots of 1.0 × 10^−10^–1.0 × 10^−2^ M standard MTF solution were pipetted into a 50-mL beaker, and the prepared sensors in conjunction with the Ag/AgCl reference electrode were submerged in this solution. The potential was measured and recorded in mV. The slope of the calibration curves was calculated after the electrode potential was plotted against −log concentration of the examined drug.

### 2.11. Optimizing the Condition of Potential Reading

The impact of pH on the potential of the prepared sensors was measured. The reference electrode Ag/AgCl was connected to the coated wire sensor. About 50 mL aliquots of the drug solution (1.0 × 10^−5^ M or 1.0 × 10^−4^ M) were added to a 100 mL beaker and the two electrodes were immersed in it. Potential measurements corresponding to various pH values were then recorded. The pH-mV was measured and plotted after small amounts of 0.1 M HCl were added to the solution in order to first acidify it. Small amounts of 0.1 M NaOH were then added to progressively raise the pH [43].

The separation solution approach was used to determine the selectivity coefficient, and the following equation (1) was applied:

(1) $$\begin{array}{c} \text{Log }K_{{MTF.J}^{z+}}^{pot}=\frac{\left(E_{2}–E_{1}\right)}{S}+\log\left[\text{MTF}\right]‐{\log\left[J^{z+}\right]}^{1/z}, \end{array}$$

where *E*_1_ is the electrode potential of drug solution in 1.0 × 10^−3^ M, *E*_2_ is the potential of the electrode of interfering species in 1.0 × 10^−3^ M of the interferent ion J^+^, and S is the slope of the calibration graph. However, the proposed sensors' selectivity toward interfering ingredients, such as some common cations, amino acid, sugars, related organic analytes (guanidine, cycloguanil, synthalin, and galegine), and formulated additives, was studied [44].

### 2.12. Analysis of MET in Metfor® Tablets

To prepare 1.0 × 10^−2^ M standard solution, ten tablets of Metfor® (500 mg/tablet) were ground to a fine powder. An exact quantity equivalent to 0.13 g was then dissolved in distilled water. Serial dilutions were done to prepare various concentrations of MET within the range of 1.0 × 10^−10^–1.0 × 10^−2^ M.

## 3. Results and Discussion

The synthesis process of Al_2_O_3_ and NiO nanoparticles was carried out under certain optimized conditions. The number of nanoparticles produced was significantly influenced by the amount of plant extract. The absorption pattern changed significantly when the amount of plant extract was increased. The use of 20 mL of plant extract showed the highest absorbance at 290 nm and 400 nm for the Al_2_O_3_ and NiONPs, respectively. In addition, a pH of 11 and a reaction time of 30 minutes at room temperature were chosen.

### 3.1. Characterization of Al_2_O_3_ and NiO Nanoparticles

**Figure 2:**
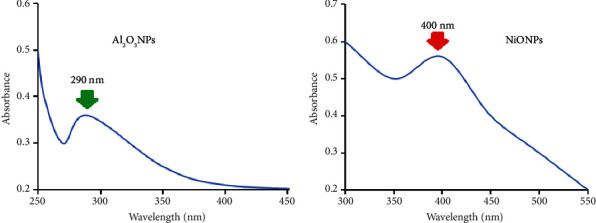
UV-vis spectra of the synthesized: (a) Al_2_O_3_NPs and (b) NiONPs using the millet seed extract.

The obtained Al_2_O_3_NPs and NiONPs were examined using a variety of spectroscopic and microscopic techniques. Using UV-vis spectroscopy, the optical properties of each metal oxide nanoparticle were studied. The absorption spectra in Figures 2(a) and 2(b) showed absorption peaks at 290 nm for Al_2_O_3_NPs and 400 nm for NiONPs.

The band gap energy of the prepared Al_2_O_3_NPs and NiONPs was calculated from the following formula:

(2) $$\begin{array}{c} \alpha hʋ=A{\left(hʋ‐\text{Eg}\right)}^{n}, \end{array}$$

where *α* is the absorption coefficient, Eg is the band gap energy, *h* is the Planck constant (6.626 × 10^–34^ J·s), *n* is 1/2 or 2 for direct or indirect transition, and (hʋ) is the photon energy (eV). The predicted band gaps for the Al_2_O_3_NPs and NiONPs were 4.28 eV and 3.1 eV, respectively.

To validate the role of bioactive compounds in the green synthesis of metal oxide nanostructures, the absence or presence of phytochemicals in the millet extract has been tested using preliminary chemical tests. According to the previous report [45], various reagents were used including, for flavonoids (sodium hydroxide and hydrochloric acid), alkaloids (Dragendroff reagent, HCL), saponin (Foam test), tannins (ferric chloride 1% solution), terpenoids (Salkowski's test), phenols (bromine water, white ppt), proteins (Biuret test, copper sulfate solution), and carbohydrates (Tollen's test). The obtained findings are summarized in Table 1.

**Figure 3:**
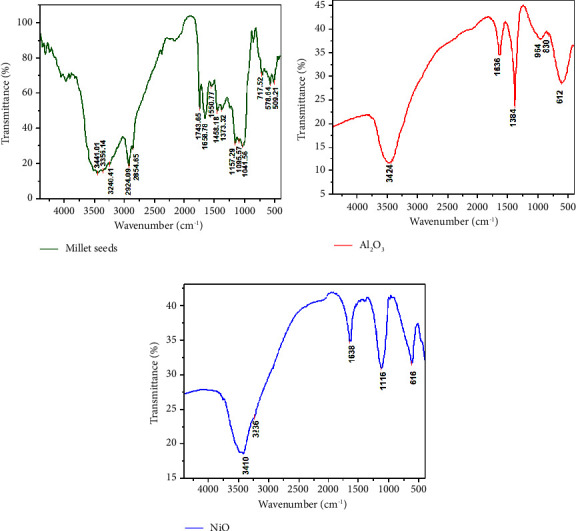
FT-IR spectra of (a) millet seed extract, (b) Al_2_O_3_NPs and (c) NiONPs measured at the wavenumber range 4000–400 cm^−1^.

Due to the presence of phenolic compounds, such as ferulic acid, which is the main bound phenolic compound, the FT-IR analysis of the millet seed extract (Figure 3(a)) revealed different functional groups at 3441, 3356, and 3240 cm^−1^ for O-H stretching vibration. In addition, 2924 and 2854 cm^−1^ are related to asymmetric and symmetric C-H stretching of alkane. The absorption band at 1743 cm−1 is for C=O stretching of esters while the absorption bands at 1658 and 1550 cm−1 are for asymmetric C=O stretching vibration of aqueous carboxylate. The appeared bands at 1458 and 1373 cm^−1^ are related to NO_2_ compounds. However, the noticed bands in the range from 1157 to 1041 cm^−1^ are associated with C-O stretching of polysaccharide skeleton in millet carbohydrates. The bands of 717−509 cm^−1^ are for stretching vibration of alkyl-halide compounds [46].

The spectra of Al_2_O_3_NPs are shown in Figure 3(b). The broad peaks at 3752.52 cm^−1^ and 3471.56 cm^−1^ are for OH stretching, peaks at 1635.93 cm^−1^ and 1544.23 cm^−1^ are for O-H stretching and bending vibration of absorbed water, peaks at 1383.93 cm^−1^ and 961.87 cm^−1^ could be attributed to O-H bending and C=C bending, the peak at 832.95 is for Al-O vibration, and the broad absorption peak at 608 cm^−1^ is assigned to Al-O-AL stretching vibration mode [47] in the FT-IR. The spectra of NiO are shown in Figure 3(c). The broad peaks at 3489.44 cm^−1^ and 3419.07 cm^−1^ are for -OH stretching, the peaks at 1637.17 cm^−1^ and 1401.39 cm^−1^ are for C=C and C=O stretching, the peak at 1116 cm^−1^ could be attributed to C-O stretching, and the broad absorption peak at 616 cm^−1^ is assigned to the Ni-O vibration mode [48].

**Figure 4:**
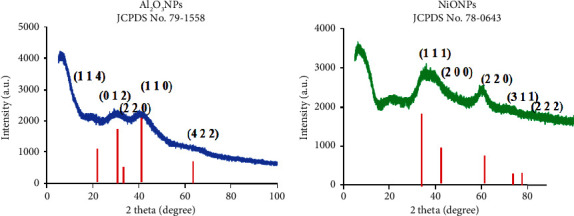
XRD patterns of (a) Al_2_O_3_NPs and (b) NiONPs synthesized using the millet seed extract.

The XRD analysis was used to study the crystalline shape and determine the crystallite size of the synthesized metal oxide nanostructures. The XRD pattern of Al_2_O_3_NPs (Figure 4(a)) shows remarkable peaks at 2*θ* = 22.9°, 29.4°, 31.9°, 39.0°, 55.6°, and 64.9° assigned to planes (1 1 4), (0 1 2), (2 2 0), (1 1 0), and (4 2 2), respectively. The obtained findings are matched to JCPDS Card No. 79–1558 [49]. The XRD pattern of NiO (Figure 4(b)) shows different peaks at (2*θ*) of NiONPs at 37.04°, 43.04°, 62.49°, 74.93°, 78.89, and 94.38 assigned to planes (1 1 1), (2 0 0), (2 2 0), (3 1 1), and (2 2 2), respectively. The obtained findings are matched to JCPDS Card No. 78–0643 [50]. The grain size of the Al_2_O_3_ and NiO crystallites was determined using the Debye–Scherrer formula:

(3) $$\begin{array}{c} D=\frac{0.94\lambda }{\beta \mathrm{Cos}\theta }, \end{array}$$

where *D* is the crystal size, *λ* = 1.54060 is the wavelength of the radiation, *β* is the line broadening at half the maximum intensity, and *θ* is the Bragg angle of the X-ray diffraction peak. The average crystallite size of Al_2_O_3_ and NiO was 17.7 nm and 20.2 nm, respectively.

**Figure 5:**
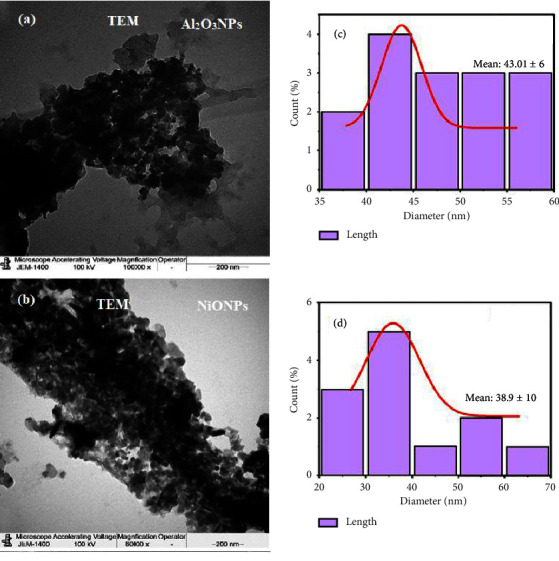
(a, b) TEM images and (c, d) average particle size using J-images software.

TEM is a technique that provides much higher resolution than light-based imaging methods by imaging a nanoparticle sample with an electron beam. The TEM examination revealed details about the surface morphology, agglomeration, and particle size. The TEM images of Al_2_O_3_NPs and NiONPs showed that the particles are fairly uniformly distributed, polydisperse, and spherical (Figures 5(a) and 5(b)) with an average particle size of 43.01 ± 6 and 38.9 ± 10 nm (Figures 5(c) and 5(d)).

**Figure 6:**
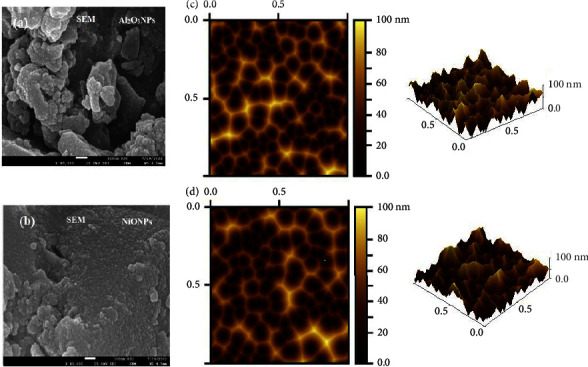
(a, b) SEM images and (c, d) AFM images of Al_2_O_3_NPs and NiONPs with 3D images.

The primary application of the versatile and advanced field emission scanning electron microscope (FISEM) is the study of material surface phenomena. Numerous qualitative details about a material, including its composition, topography, morphology, and crystallographic properties, can be obtained using a FISEM. Stated differently, it furnishes data regarding the size, form, and distribution of the particles on the sample's surface, in addition to the surface properties and texture [51]. The characteristics of adsorbents that have the largest effects on their capacity to absorb biomolecules are their surface area, morphology, charge, and state of aggregation. The morphology of the synthesized metal oxide nanoparticles was investigated using the FESEM. The micrographs showed that each Al_2_O_3_NP and NiONP had a spherical morphology with particle sizes ranging from 50 to 100 nm (Figures 6(a) and 6(b)). Large cavities and pores along with an uneven, porous, and heterogeneous surface morphology are visible in the nanocomposite's 30,000x magnification FESEM image. There are some visible micropores in addition to the mesopores. Because of its greater surface area, NCS's heterogeneity, pores, and voids greatly enhance the material's adsorption capacity. The size and three-dimensional profile of the synthesized nanomaterials were verified by AFM analysis of their surface characteristics. The AFM images (Figures 6(c) and 6(d)) demonstrate a well-defined spherical shape with a solid, dense structure and an average size of 100 nm for the particles.

### 3.2. The Fabricated Sensor Behavior

MTF reacts with PTA to form a stable MTF-PT ion pair complex, which was soluble in an organic solvent such as THF but insoluble in water. The addition of the active components with (*o*-NPOE) acting as a solvent mediator with the presence of PVC was used in conventional and modified sensors [52]. Critical response characteristics of the fabricated electrodes over the concentration range 1.0×10^−6^–1.0 × 10^−2^ and 1.0 × 10^−10^–1.0×10^−2^ M for conventional and modified sensors were studied, and the results are summarized in Table 2.

**Figure 7:**
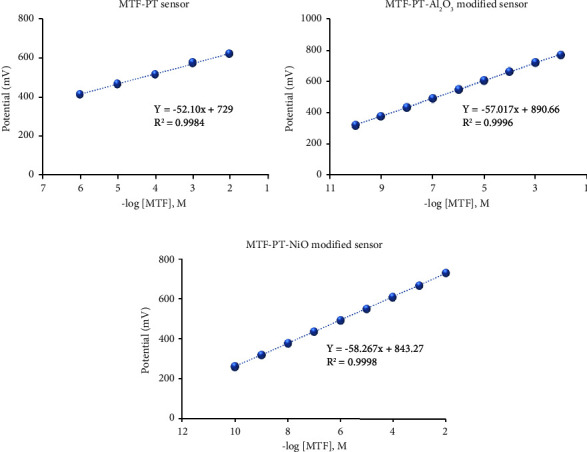
Calibration graphs of (a) the MTF-PT conventional sensor, (b) the MTF-PT-Al_2_O_3_-modified sensor, and (c) the MTF-PT-NiO-modified sensor.

The fabricated sensors gave Nernstian responses with slopes of 52.1 ± 0.5, 57.01 ± 0.4, and 58.27 ± 0.7 for MTF-PT, MTF-PT-Al_2_O_3_, and MET-PT-NiO, respectively (Figures 7(a)–7(c)). The suggested sensors have fast dynamic response times of 45, 20, and 30 s and are used for a period of 27, 45, and 35 days for MTF-PT, MTF-PT-Al_2_O_3_, and MTF-PT-NiO, respectively, without any significant change in parameters. The results showed that when compared to the conventional and the modified sensors, those enhanced with metal oxide nanoparticles had quick response times and good stability. This could be caused by the addition of nanoparticles to sensors. They have physicochemical properties not found in the bulk material. These nanoparticles improved interactions with targets in test solutions due to their higher surface-to-volume ratio [53].

**Figure 8:**
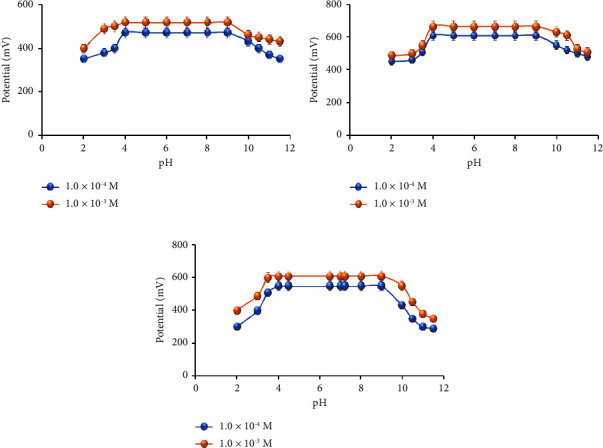
The pH effect on the response of (a) the MTF-PT-modified sensor, (b) the MTF-PT-Al_2_O_3_-modified sensor, and (c) the MTF-PT-NiO-modified sensor.

The pH effect of sensors on the potential was investigated to determine the appropriate pH range for determining MTF. The result of conventional and modified sensors concluded that they were practically independent in the range of 4–9 and could be safely used for MTF determination. Potential-pH curves for MTF concentration were created, as shown in Figures 8(a)–8(c). Below pH 4, the potential dropped as the acidity of the analyte increased, which could be attributed to the membrane extraction of H^+^ ions. The decrease in the electrode response at pH levels greater than 9 may be attributed to the rise in OH^−^ concentrations.

One of the most important aspects of an ion-selective electrode is undoubtedly its selectivity behavior, which determines the feasibility of a trustworthy measurement in the target sample. For a number of inorganic cations, sugars, amino acids, and related compounds, the separated solution method [54] and the matched potential method [55] were used to determine the selectivity coefficients for MTF cations. How different substances affect the response of MTF-PT, MTF-PT-Al_2_O_3_, and MTF-PT-NiO-coated wire membrane sensors was investigated. The selectivity of the prefabricated sensors was tested by measuring the potentiometric interference of inorganic cations such as Na^+^, Fe^3+^, Cr^3+^, Ag^+^, Ca^2+^, K^+^, Mg^2+^, and Co^2+^; sugars; and amino acids. The physicochemical properties of the ion exchange process at the membrane determine the selectivity of a membrane sensor based on ion pairs. This selectivity could result from the free energy transfer of the MTF^+^ ions between the membrane and the surrounding medium. The data show that the proposed electrodes exhibit a high degree of selectivity for MTFs. Due to the different permeability and mobility of the inorganic cations with respect to the MTFs, they do not interfere, with the so-called Hofmeister selectivity sequence [56]. The degree of correspondence between the locations of lipophilicity sites in two competing species on the bath solution side and those in the receptor of the ion exchanger determines the mechanism of selectivity, which is primarily based on stereospecificity and the electrostatic environment [57]. The results (Table 3) showed that there was no interference between amino acids and sugars.

The selectivity of the produced sensors was investigated with guanidine and other related compounds. No interferences were observed, which can be attributed to the electroactive sites in the membrane (MTF-PT). However, the addition of metal oxide nanoparticles with a large surface area and high dielectric constant increases the conductivity of the sensor and thus improves the selectivity and sensitivity.

### 3.3. Quantification of Metformin Hydrochloride

The created sensors were used to detect MTF in its bulk powder. The results were obtained using the direct calibration method and were expressed as % recoveries. The analysis's results, which used the suggested electrodes, revealed mean percentage recoveries of 98.87 ± 0.72, 99.60 ± 0.34, and 99.45 ± 0.40 for MTF-PT, MTF-PT-Al_2_O_3_NPs, and MTF-PT-NiONPs, respectively (Table 4).

These results showed the ultra-sensitivity of the MTF-PT-Al_2_O_3_NP- and MTF-PT-NiONP-modified sensors. The special physical and chemical properties of the metal oxide nanoparticles in use improved the conductivity and sensitivity of the modified electrodes for the detection of the selected drug.

### 3.4. Method Validation

The proposed method was validated according to IUPAC recommendations [58]. Wide linear relationships were displayed by the designed sensors over 1.0×10^−6^–1.0 × 10^−2^ M for the conventional sensor, in comparison with 1.0 × 10^−10^–1.0 × 10^−2^ M for the modified sensors. The regression equations were *E*_mv_ = (52.1 ± 0.5) log [MTF] + 729 for the MTF-PT conventional sensor and *E*_mv_ = (57.01 ± 0.4) log [MTF] + 890.66 and *E*_mv_ = (58.27 ± 0.7) log [MTF] + 843.27 for MTF-PT-Al_2_O_3_NP- and MTF-PT-NiONP-modified sensors, with correlation coefficients *r* = 0.998, 0.999, and 0.999 for the respective sensors stated, respectively. All sensors' lower limits (LOD) of detection were recorded after the slope's potential reading dropped by 17.9 mV. The obtained results were found to be 5.0 × 10^−7^, 5.0 × 10^−11^, and 5.0 × 10^−11^ M. The dielectric constant of NiO is *ε* = 9.1, and the dielectric constant of aluminum oxide is *ε* = 9-10; the two metal oxides have almost the same conductivity constant, which is why both sensors modified with Al_2_O_3_ and NiO nanoparticles have the same lower detection limit for MTF analysis.

Nine concentrations were used to test the method's accuracy, and the mean percentage recoveries were calculated as 98.98 ± 0.57, 99.69 ± 0.38, and 99.55 ± 0.53 for the above-mentioned sensors, respectively. Additionally, the intermediate precision was evaluated via the inter-day and intra-day assay, and the percentage relative standard deviation (% RSD) was calculated. The % RSD for the MTF-PT-Al_2_O_3_NP-modified sensor was 0.27% and 0.41%, and for the MTF-PT-NiONP-modified sensor, it was 0.26% and 0.21%. All results are less than 2%, showing a highly precise technique (Table 5).

Borate buffer with a pH of 9 ± 0.5 was used to ensure the method's robustness. The percentage recovery for the conventional sensor was 99.44 ± 0.4%, the modified MTF-PT-Al_2_O_3_NPs sensor was 99.65 ± 0.2%, and the modified MTF-PT-NiONPs sensor was 99.77 ± 0.3%. Another test was performed to ensure the ruggedness of the suggested method by using a different model of pH meter (Jenway-3510). The measured mean percentage recoveries were 99.77 ± 0.2%, 99.85 ± 0.1%, and 99.50 ± 0.1% for the previously mentioned sensors. The results showed good agreement with those obtained using the proposed technique with no significant changes observed.

### 3.5. Determination of MTF Hydrochloride in Tablets

To quantify the metformin hydrochloride in its pharmaceutical form Metfor® (500 mg/tablet), the fabricated MET-PT, MET-PT-Al_2_O_3_, and MET-PT-NiO sensors were used. The potential readings were recorded for different concentrations of MTF samples, and the recovery percentage was calculated. The results were 99.12 ± 0.62, 99.57 ± 0.46, and 99.35 ± 0.60 for the above-mentioned sensors, respectively (Table 6).

## 4. Conclusion

The proposed potentiometric study was conducted by fabricating coated wire sensors enriched with aluminum oxide and nickel oxide nanoparticles. The potential readings of MTF-PT-Al_2_O_3_NP- and MTF-PT-NiONP-modified sensors were compared with the MTF-PT conventional sensor. For the quantification of diabetic metformin hydrochloride due to their sensitivity and selectivity, the created sensors proved to be effective and superior to the conventional sensors. In addition, the use of Al_2_O_3_NPs and NiONPs as electro-improved materials increased the sensitivity of the sensors and made it easier to identify the drug under investigation with a low limit and over a wide concentration range. The fabricated electrodes can be applied for the metformin routine analysis in pharmaceutical industries, research laboratories, and hospitals.

## Figures and Tables

**Table 1:** Qualitative determination for preliminary phytochemicals in the millet extract.

Phytochemicals	Chemical test	Positive indication sign
Flavonoids	Sodium hydroxide few drops and few drops of hydrochloric acid	Deep yellow color was removed by few drops of hydrochloric acid
Alkaloids	Dragendorff reagent	Orange or red precipitate
Tannins	Ferric chloride (1%)	Blackish blue color
Saponin	Foam test	Foam more than 1 cm
Terpenoids	Salkowski's test	Reddish-brown precipitate
Phenols	Bromine water	White precipitate
Proteins	Biuret test, copper sulfate solution	
Carbohydrates	Tollen's reagent	Silver mirror

**Table 2:** Electrochemical response characteristics of MTF-PT conventional and MTF-PT-Al_2_O_3_-modified and MET-PT-NiO-modified sensors.

Parameter	MTF-PT conventional sensor	MTF-PT-Al_2_O_3_-modified sensor	MTF-PT-NiO-modified sensor
Slope (mV decade^−1^)	52.1 ± 0.5	57.01 ± 0.4	58.27 ± 0.7
Intercept (a)	729	890.66	843.27
Correlation coefficient (r)	0.9992	0.9998	0.9999
Linear range (M)	1.0×10^−6^‐1.0 × 10^−2^	1.0 × 10^−10^‐1.0 × 10^−2^	1.0 × 10^−10^‐1.0 × 10^−2^
LOD (M)	5.0 × 10^−7^	5.0 × 10^−11^	5.0 × 10^−11^
Response time, (s)	45	20	30
Working pH range	4‐9	4‐9	4‐9
Lifetime (day)	27	45	35
Temperature (°C)	25	25	25
Accuracy (%)	98.98 ± 0.57	99.69 ± 0.38	99.55 ± 0.53

**Table 3:** Selectivity coefficient (*K*_Pot_ MTF^+^) of conventional MTF-PT and MTF-PT-Al_2_O_3_-modified and MTF-PT-NiO-modified sensors using 1.0 × 10^−3^ M of MTF solution.

Interferences	*K*_pot_ MTF-PT	*K*_pot_ MTF-PT-Al_2_O_3_	*K*_pot_ MTF-PT-NiO
Na^+^	1.3 × 10^−3^	1.4 × 10^−4^	9.3 × 10^−3^
Fe^3+^	2.5 × 10^−3^	1.6 × 10^−3^	5.1 × 10^−3^
Cr^3+^	5.0 × 10^−3^	5.1 × 10^−3^	1.9 × 10^−3^
Ag^+^	3.2 × 10^−3^	2.2 × 10^−4^	1.7 × 10^−3^
Ca^2+^	3.8 × 10^−3^	5.7 × 10^−3^	3.9 × 10^−3^
K^+^	7.9 × 10^−3^	1.9 × 10^−4^	1.6 × 10^−4^
Mg^2+^	1.1 × 10^−3^	4.5 × 10^−4^	3.2 × 10^−4^
Co^2+^	1.4 × 10^−3^	2.7 × 10^−3^	1.0 × 10^−3^
Serine	1.3 × 10^−3^	5.5 × 10^−3^	5.0 × 10^−4^
Glycine	2.1 × 10^−3^	1.3 × 10^−4^	1.6 × 10^−3^
Starch	1.9 × 10^−3^	2.3 × 10^−4^	1.0 × 10^−3^
Guanidine	1.1 × 10^−3^	9.2 × 10^−4^	8.2 × 10^−4^
Cycloguanil	2.3 × 10^−3^	1.8 × 10^−4^	9.3 × 10^−4^
Synthalin	5.6 × 10^−3^	5.9 × 10^−4^	6.5 × 10^−4^
Galegine	4.2 × 10^−3^	3.6 × 10^−4^	7.4 × 10^−4^

**Table 4:** The results of the MTF determination in bulk powder using MTF-PT, MTF-PT-Al_2_O_3_NP-modified, and MTF-PT-NiONP-modified sensors.

	MTF-PT conventional sensor			MTF-PT-Al_2_O_3_NP-modified sensor			MTF-PT-NiONP-modified sensor		
	Test^*∗*^ solution	Found^*∗*^	% recovery	Test^*∗*^ solution	Found^*∗*^	%Recovery	Test^*∗*^ solution	Found^*∗*^	% recovery
Statistical analysis	6	5.99	99.83	10	9.96	99.60	10	9.95	99.50
	5	4.96	99.20	8	7.99	99.87	8	7.96	99.50
	4.3	4.20	97.76	6	5.98	99.66	6	5.94	99.00
	4	3.97	99.25	4	4.00	100.00	4	3.99	99.75
	3	2.96	98.67	3	2.97	99.00	3	2.97	99.00
	2	1.97	98.50	2	1.99	99.50	2	2.00	100.00

Mean ± SD	98.87 ± 0.72			99.60 ± 0.34			99.45 ± 0.40		

*n*	6			6			6		

Variance	0.52			0.12			0.16		

% SE^*∗∗*^	0.29			0.14			0.16		

% RSD	0.73			0.34			0.40		

^*∗*^Test and found solutions −log conc. (mol L^−1^) ^*∗∗*^SE (% Error) = %RSD/√n.

**Table 5:** Intraday and interday assays of MTF solution using MTF-PT-Al_2_O_3_NP-modified and MTF-PT-NiONP-modified sensors.

	MTF-PT-Al_2_O_3_NP-modified sensor			MTF-PT-NiONP-modified sensor		
	Test sample	Found	% recovery	Test sample	Found	% recovery
Intraday	10	10.00	100.00	10	9.99	99.90
	8	7.96	99.50	8	7.95	99.38
	6	5.99	99.93	6	5.98	99.67

Mean ± SD	99.81 ± 0.27			99.65 ± 0.26		

*n*	3			3		

SE%	0.16			0.15		

RSD%	0.27			0.26		

Interday	10	9.99	99.90	10	9.95	99.50
	8	7.94	99.25	8	7.94	99.25
	6	6.00	100.00	6	5.98	99.67

Mean ± SD	99.72 ± 0.41			99.47 ± 0.21		

*n*	3			3		

SE%	0.24			0.12		

RSD%	0.41			0.21		

^*∗∗*^SE (% Error) = %RSD/√n.

**Table 6:** The results of the MTF determination in its dosage forms using MTF-PT, MTF-PT-Al_2_O_3_NP-modified, and MTF-PT-NiONP-modified sensors with respect to the previously reported results.

	MTF-PT conventional electrode			MTF-PT-Al_2_O_3_NP- modified electrode			MTF-PT-NiONP- modified electrode			Reference method [59]
	Test sample	Found	% recovery	Test sample	Found	% recovery	Test sample	Found	%Recovery	
Statistical analysis	6	5.98	99.66	10	10.00	100.00	10	9.96	99.60	
	5	4.97	99.40	8	7.96	99.75	8	7.94	99.25	
	4.3	4.25	98.83	6	5.94	99.00	6	5.96	99.33	
	4	3.98	99.50	4	4.00	100.00	4	3.93	98.25	
	3	2.98	99.33	3	2.99	99.67	3	2.99	99.67	
	2	1.96	98.00	2	1.98	99.00	2	2.00	100.00	

Mean ± SD	99.12 ± 0.62			99.57 ± 0.46			99.35 ± 0.60			99.25 ± 0.72

*n*	6			6			6			6

Variance	0.38			0.21			0.36			0.52

%SE	0.25			0.19			0.24			0.29

%RSD	0.63			0.46			0.60			0.74

*t*-test	0.337 (2.228)^*∗*^			0.922 (2.228)^*∗*^			0.266 (2.228)^*∗*^			

*F*-test	1.37 (5.05)^*∗*^			2.48 (5.05)^*∗*^			1.44 (5.05)^*∗*^			

## Data Availability

All data supporting this study are included within the text.
